# Potential Benefit of Intravenous Iron Monotherapy for Grade 2–3 Chemotherapy-Induced Anemia Irrespective of Transferrin Saturation Levels

**DOI:** 10.7759/cureus.99274

**Published:** 2025-12-15

**Authors:** Tomoko Ueda, Hiroshi Tsubamoto, Haruka Honda, Sachiyo Narita, Yumi Takimoto, Maiko Iwamoto, Maiko Korosue, Yu Wakimoto, Tomoyuki Sasano, Seiji Mabuchi

**Affiliations:** 1 Department of Obstetrics and Gynecology, School of Medicine, Hyogo Medical University, Nishinomiya, JPN

**Keywords:** chemotherapy-induced anemia, ferric carboxymaltose, ferric derisomaltose, iv iron monotherapy, transferrin saturation

## Abstract

Background

High-dose intravenous (IV) iron monotherapy has been evaluated for chemotherapy-induced anemia (CIA), but prior studies mainly focused on patients with hemoglobin (Hb) levels around 10 g/dL. Its efficacy in more severe anemia and the predictive value of transferrin saturation (TSAT) remain unclear.

Methodology

We retrospectively reviewed cancer patients who underwent chemotherapy and received intravenous ferric carboxymaltose or ferric derisomaltose. The primary endpoint was an Hb increase of ≥1 g/dL within one month, with patients classified as responders, and response rates were compared using a TSAT cutoff of 20%. Hb measurements obtained after delays or discontinuation of chemotherapy were censored.

Results

Twenty patients (median pretreatment Hb, 8.5 g/dL; range, 6.9-9.7 g/dL) were included in this study. Eight patients (40%) achieved an Hb increase of ≥1 g/dL within one month. Response rates were 36% in patients with TSAT <20% and 40% in those with TSAT ≥20%. No significant association was observed with serum ferritin levels or iron dose.

Conclusions

IV iron monotherapy has potential benefit in a subset of patients with grade 2-3 CIA. Routine measurement of TSAT or ferritin may not be necessary in daily practice, particularly for patients who are ineligible for erythropoiesis-stimulating agent therapy.

## Introduction

Anemia occurs in approximately 40% of cancer patients undergoing chemotherapy [1]. It significantly impairs quality of life through symptoms such as palpitations, fatigue, and lightheadedness, leading to limitations in daily activities at home and work. Anemia may also cause chemotherapy dose reductions or delays, thereby decreasing relative dose intensity and potentially compromising treatment efficacy. According to Western clinical guidelines, including those issued by the European Society for Medical Oncology (ESMO) [2] and the National Comprehensive Cancer Network (NCCN) [3], iron supplementation is recommended for patients with mild anemia, whereas erythropoiesis-stimulating agents (ESAs) are the mainstay of treatment for patients with hemoglobin (Hb) levels below 10 g/dL. As an exception, iron monotherapy is also considered for patients with Hb <10 g/dL when transferrin saturation (TSAT) is low, suggesting absolute iron deficiency.

However, the use of ESAs is associated with clinically relevant adverse events, including thromboembolic complications and potential negative effects on cancer prognosis. In Japan, ESAs are not approved for the treatment of anemia in patients with active malignancy. Consequently, for patients with Hb <10 g/dL, treatment options are essentially limited to red blood cell transfusion or intravenous (IV) iron supplementation [4]. While transfusion provides rapid correction of anemia, it is associated with risks such as infection, transfusion reactions, and immunomodulation. Therefore, IV iron represents a potentially safer and more sustainable therapeutic alternative for this patient population. Despite this clinical need, the efficacy of IV iron monotherapy in patients with more severe chemotherapy-induced anemia (CIA) (Hb <10 g/dL) has not been adequately established. In addition, it remains unclear whether iron metabolic parameters, particularly TSAT, can reliably predict the hematological response to IV iron in this setting. Given these unresolved clinical issues, the present study aimed to evaluate the efficacy of IV iron monotherapy in patients with CIA and Hb <10 g/dL, with a special focus on whether baseline TSAT and related iron parameters can serve as predictors of treatment response.

## Materials and methods

This was a retrospective study of cancer patients undergoing chemotherapy who received IV iron (ferric carboxymaltose (FCM) or ferric derisomaltose (FDI)) between October 2024 and July 2025 at the Hyogo Medical University Hospital, Hyogo, Japan. The study was approved by the Institutional Review Board of Hyogo Medical University (approval number: 5126) and was conducted in accordance with the Declaration of Helsinki.

Study population

Eligible patients were those who received high-dose IV iron at doses of 500 mg of FCM or 1000 mg of FDI administration, with the maximum cumulative dose set at 1500 mg and 2000 mg, respectively, according to the approved labeling. IV iron was primarily administered to patients with CIA presenting with Hb levels <10 g/dL accompanied by anemia-related symptoms that interfered with daily activities, or to asymptomatic patients with Hb levels <8 g/dL. The choice of FCM or FDI and the maximum administered doses were determined by the attending physicians. Patients who received IV iron at the initiation of chemotherapy were included. To minimize bias from Hb recovery related to bone marrow recovery after chemotherapy-induced myelosuppression, subsequent Hb measurements were censored and excluded from the efficacy analysis when chemotherapy was postponed or discontinued. Administration of IV iron after the day of the last cycle of chemotherapy was also excluded.

Study endpoints

The primary endpoint was an increase in Hb of ≥1 g/dL at any point within one month after the initial administration of IV iron according to the previous report [5], and treatment response stratified by TSAT using a cutoff value of 20% and serum ferritin levels using a cutoff value of 500 ng/mL. Secondary endpoints included maintenance of Hb levels without further anemia progression, comparison of response by dosage, and incidence of side effects. The TSAT cutoff of 20% was based on the ESMO and NCCN guidelines, while the ferritin cutoff was derived from the NCCN definition of functional iron deficiency. From a clinical perspective, prevention of further Hb decline during chemotherapy was considered clinically meaningful; therefore, stabilization of Hb levels following IV iron administration was also included among the secondary endpoints.

Data analysis

Statistical analyses were performed using Fisher’s exact test with GraphPad Prism 9 software (Dotmatics, Boston, Massachusetts, United States). A p-value <0.05 was considered statistically significant.

## Results

**Figure 1:**
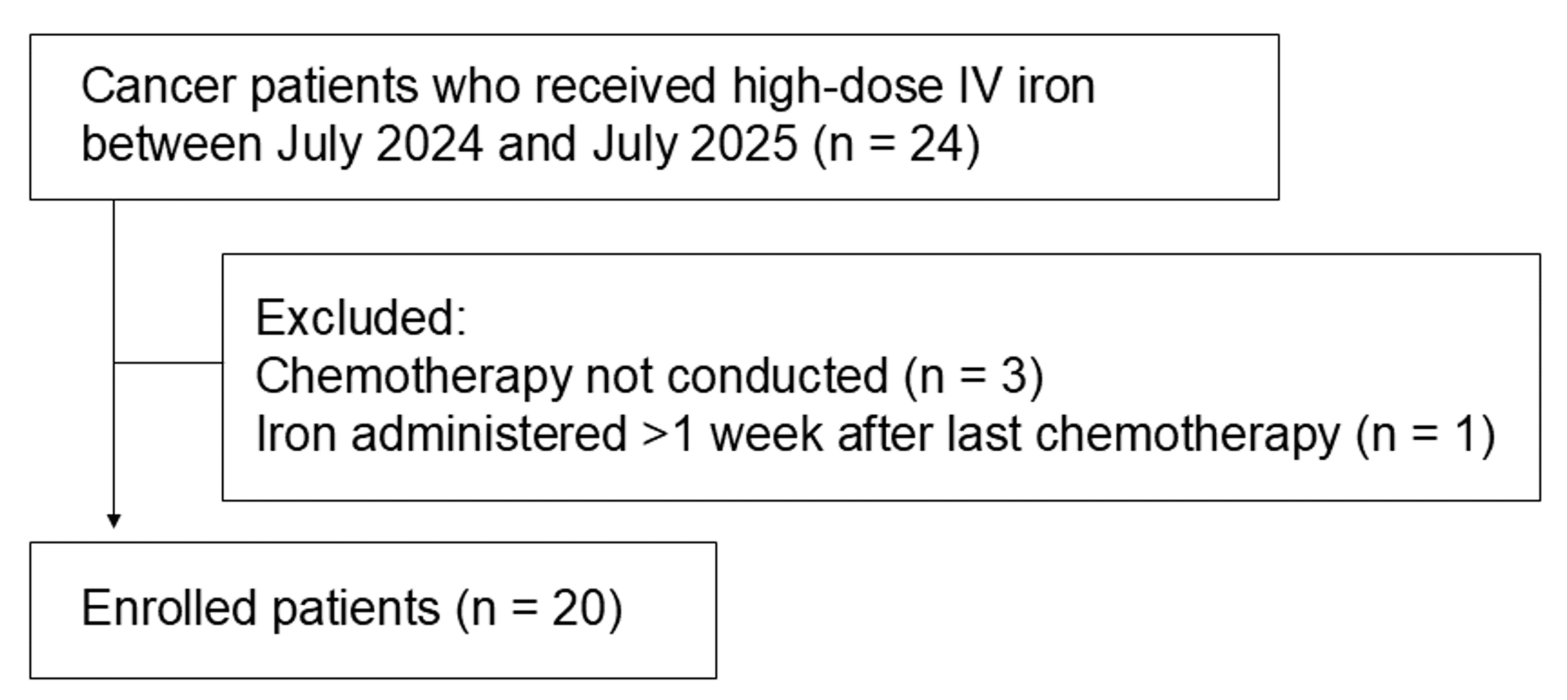
Patient flow diagram Cancer patients who received high-dose intravenous iron between October 2024 and July 2025 were identified (n = 24). Patients were excluded if chemotherapy was not conducted (n = 3) or if iron was administered after the last chemotherapy (n = 1), leaving 20 patients for further analysis.

During the study period, IV iron therapy was administered to 24 cancer patients, of whom 21 were undergoing chemotherapy. One patient received IV iron one week after the last chemotherapy cycle. Ultimately, 20 patients met the inclusion criteria and were included in the efficacy analysis of high-dose IV iron therapy (Figure 1). Patient characteristics are summarized in Table 1. 

**Table 1:** Baseline characteristics of the 20 included patients Four patients had hemoglobin measurements but no assessments of serum iron, total iron-binding capacity, or serum ferritin. PLD, pegylated liposomal doxorubicin; PARP, poly (ADP-ribose) polymerase inhibitor; PRO-CTCAE, patient-reported outcomes version of the Common Terminology Criteria for Adverse Events; Hb, hemoglobin; TIBC, total iron-binding capacity; TSAT, transferrin saturation (serum iron/total iron-binding capacity × 100); FCM, ferric carboxymaltose; FDI, ferric derisomaltose.

Characteristics	Values
Age (years), median (range)	57 (44-76)
Cancer types, n (%)	
Ovarian cancer	13 (65%)
Cervical cancer	4 (20%)
Endometrial cancer	3 (15%)
Chemotherapy regimen, n (%)	
Paclitaxel and carboplatin based	11 (55%)
Docetaxel and carboplatin	2 (10%)
PLD and carboplatin	2 (10%)
Irinotecan	2 (10%)
Gemcitabine, cisplatin and bevacizumab	1 (5%)
PARP inhibitor	2 (10%)
Pre-treatment Hb CTCAE ver 5	
Grade 2 (Hb 8.0 to <10.0 g/dL)	14 (70%)
Grade 3 (Hb 6.5 to <8.0 g/dL)	6 (30%)
Shortness of breath or heart palpitations (PRO-CTCAE ver 1)	
Severe	4(20%)
Not severe	16(80%)
Baseline hematological parameters, median (range)	
Hb (g/dL)	8.5 (6.9-9.7)
Serum iron (μg/dL)	43 (19-159)
TIBC (μg/dL)	289 (207―574)
TSAT (%)	16 (6―61)
Ferritin (ng/mL)	288 (162―1811)
Drug administration, n (%)	
Single administration of 500 mg FCM	12 (60%)
Single administration of 1000 mg FDI	7 (35%)
Three administrations of 500 mg FCM	1 (5%)

**Figure 2:**
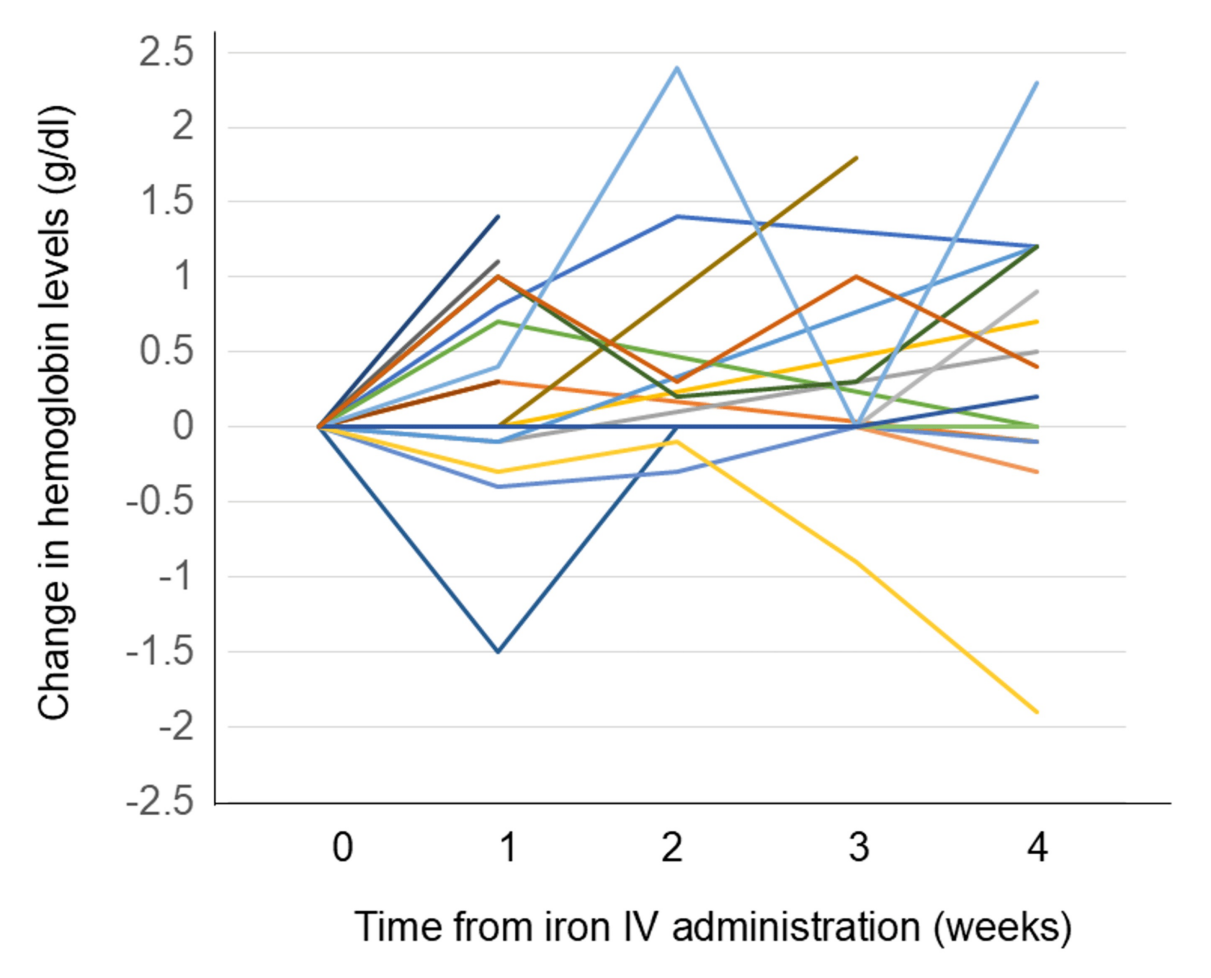
Spider plots of hemoglobin level changes after high-dose intravenous iron therapy (N = 20) Individual hemoglobin trends following ferric carboxymaltose or ferric derisomaltose administration are shown. Each line represents one patient. Eight patients (40%) achieved an increase in Hb of ≥1 g/dL, while 17 patients (85%) maintained Hb levels without any decrease. TSAT, transferrin saturation; Hb, hemoglobin.

Serum iron, total iron-binding capacity, and serum ferritin were assessed in 16 patients; the remaining four patients did not undergo these tests. Among the 16 tested, all had serum ferritin levels >100 ng/mL. Thus, none were classified as having absolute iron deficiency according to NCCN guidelines, nor did they meet criteria for oral iron administration under ESMO guidelines. Of the 20 patients, 19 received a single administration of IV iron (either 500 mg or 1000 mg). Eight of 20 patients (40.0%) (95% CI: 21.9-61.3%) achieved an increase in Hb of ≥1 g/dL, while 17 of 20 patients (85.0%) (95%CI: 64.0-94.8%) maintained stable Hb levels without any decrease following IV iron administration (Figure 2).

**Figure 3:**
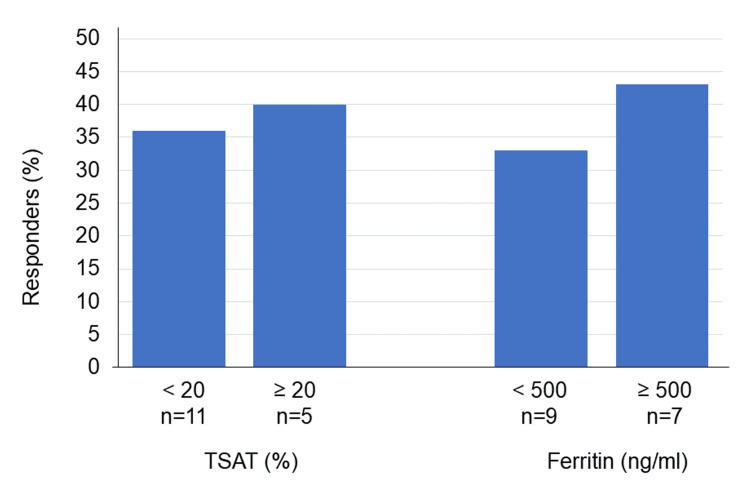
Treatment response stratified by baseline TSAT and serum ferritin (N = 16) A responder is defined as a patient whose Hb increased by ≥1 g/dL within one month of IV iron administration. There was no significant association between treatment response and TSAT using a 20% cutoff and serum ferritin levels using a 500 ng/mL cutoff (Fisher’s exact test, *p* = 0.104 and *p* = 0.063, respectively). TSAT, transferrin saturation; Hb, hemoglobin.

Among patients stratified by TSAT, four of 11 (36%) with TSAT <20% and two of five (40%) with TSAT ≥20% demonstrated an Hb increase of ≥1 g/dL within one month after IV iron administration (*p* = 0.114) (Figure 3). Similarly, three of nine (33%) patients with serum ferritin <500 ng/mL and three of seven (43%) with serum ferritin ≥500 ng/mL showed an Hb increase of ≥1 g/dL (*p* = 0.063) (Figure 3). Thus, Hb response was not significantly associated with TSAT or serum ferritin levels.

**Figure 4:**
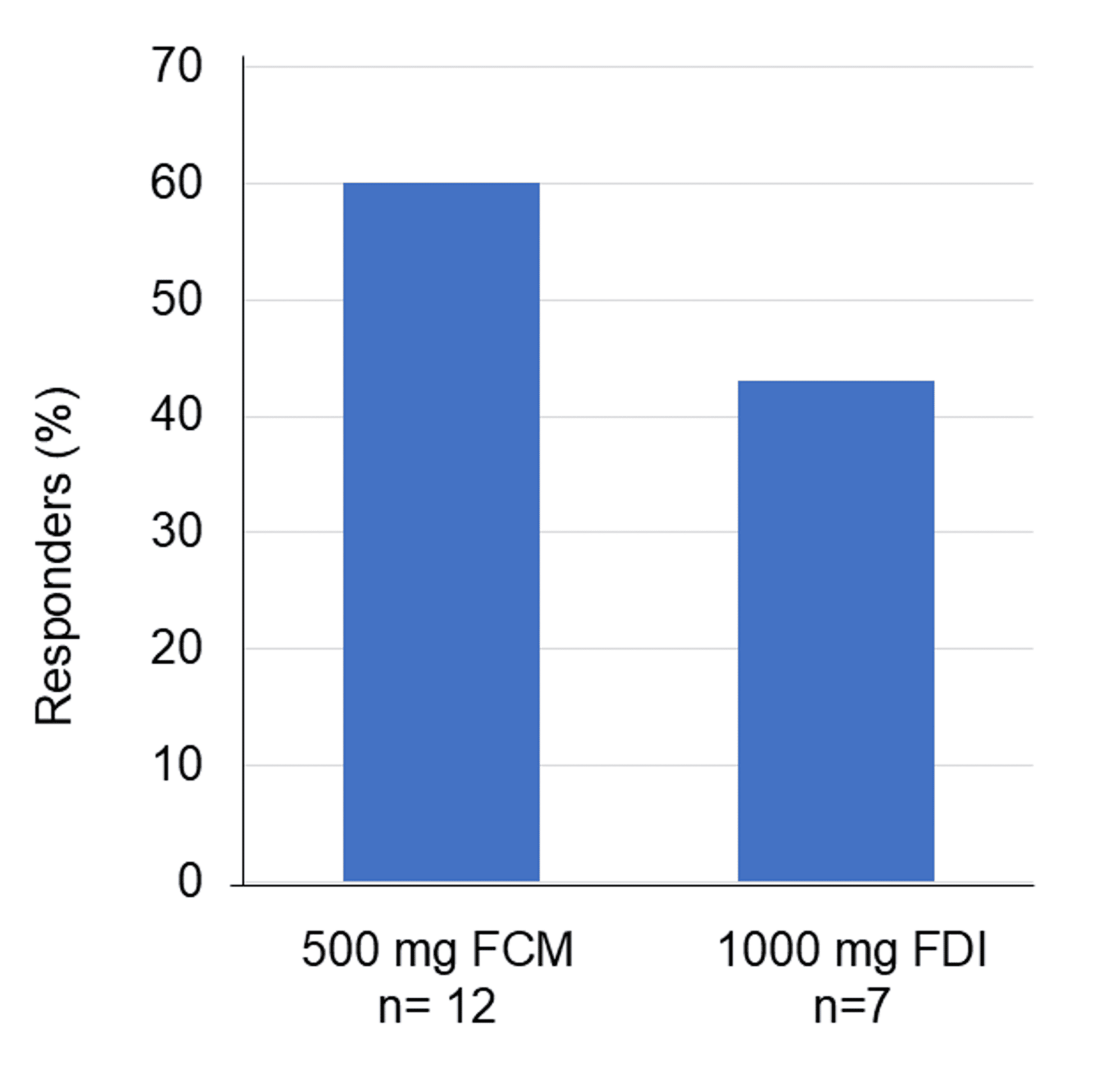
Comparison of treatment response between different iron dosage groups (N = 19) Single administration doses of 500 mg vs 1000 mg were compared. A responder is defined as a patient with a hemoglobin increase of ≥1 g/dL within one month following IV iron administration. No significant difference in response was observed between patients who received a single dose of 500 mg and those who received 1000 mg (Fisher’s exact test, *p* = 0.093). FCM, ferric carboxymaltose; FDI, ferric derisomaltose.

Five of 12 patients (42%) who received a single dose of 500 mg FCM and three of seven patients (43%) who received a single dose of 1000 mg FDI achieved an Hb increase of ≥1 g/dL. There was no significant difference in response between the 500-mg FCM and 1000-mg FDI groups (*p* = 0.099) (Figure 4). No adverse events related to IV iron administration were reported.

## Discussion

This exploratory retrospective study evaluated the clinical efficacy of IV iron monotherapy in patients with CIA and demonstrated that IV iron may be effective even in patients with Hb levels below 10 g/dL. In our cohort, neither TSAT nor serum ferritin was significantly associated with the hematological response to IV iron. These findings suggest that IV iron could be considered a potential therapeutic option for moderate to severe CIA, particularly in clinical settings where ESAs are not available for use in patients with cancer, such as in Japan. Although this study was not designed or powered to establish the predictive value of TSAT or ferritin, our results indicate that routine assessment of these parameters may not be essential when considering IV iron therapy in patients with CIA and Hb <10 g/dL.

The efficacy of IV iron monotherapy in patients with CIA and Hb levels <10 g/dL has also been suggested in a subset analysis of the IRON-CLAD randomized controlled trial [5]. In that study, eligible patients had baseline Hb levels of 8-11 g/dL, serum ferritin levels of 100-800 ng/mL, and TSAT ≤35%, and a total of 244 patients were randomized to receive FCM or placebo. Although no significant overall benefit was observed in terms of Hb improvement or symptom relief as assessed by the Functional Assessment of Chronic Illness Therapy-Fatigue scale, patients with baseline Hb <10 g/dL showed a greater increase in Hb levels with FCM compared with placebo.

NCCN guidelines recommend both IV and oral iron monotherapy for patients with absolute iron deficiency, defined as TSAT ≤20% and serum ferritin <30 ng/mL [3], whereas ESMO guidelines recommend oral iron only for patients with serum ferritin <30 ng/mL and no evidence of inflammation (C-reactive protein <5 mg/L) [2]. In the phase III PROFOUND trial comparing FDI with oral iron supplementation, only 45 of 337 patients (13%) had serum ferritin levels <30 µg/L, indicating that most patients did not meet the criteria for absolute iron deficiency [6].

In patients with cancer, proinflammatory cytokines induce hepcidin overproduction, leading to iron sequestration within macrophages and suppression of ferroportin-mediated intestinal iron absorption, which substantially limits the efficacy of oral iron therapy [7], although the biological and clinical impact of hepcidin appears to differ across tumor types [8]. In contrast, the IV administration of carbohydrate-coated iron complexes, such as FCM and FDI, bypasses intestinal absorption and enables direct uptake by macrophages, with subsequent transfer of iron to transferrin and delivery to hematopoietic tissues, thereby effectively supporting erythropoiesis [9].

In this study, iron dose was not significantly associated with treatment efficacy, in contrast to findings reported in patients with chronic kidney disease (CKD) [10], in whom higher initial doses of FCM were associated with greater Hb responses at one month. Unlike CKD, the clinical condition of patients with cancer undergoing chemotherapy is highly variable, and the progression of anemia is influenced by multiple factors, including chemotherapy-induced myelosuppression and treatment-related toxicities. These confounding factors make it difficult to isolate the therapeutic effect of IV iron alone, even though bias from bone marrow recovery was minimized by the study design. These differences between patients with cancer and those with CKD may partly explain why no significant difference was observed between the 500-mg and 1000-mg dose groups in our study.

IV iron therapy is generally well tolerated, and large observational studies and post-marketing surveillance have reported low rates of serious adverse events [11]. In the present study, no adverse events related to IV iron were identified; however, safety outcomes were assessed retrospectively based on medical record review rather than through prospective monitoring. Compared with red blood cell transfusion, which is typically indicated at lower Hb thresholds, IV iron is associated with a lower risk of infection, hospitalization, and immunological reactions. These findings suggest that early use of IV iron may represent a safer and potentially more cost-effective strategy for the management of CIA before progression to severe anemia.

This study has several limitations. First, its retrospective single-center design and small sample size limit the generalizability of the results. Moreover, the prevalence and severity of CIA may vary depending on cancer type and chemotherapy regimen, which could further influence the external validity of our findings. The chemotherapy regimens in this study have different profiles of anemia progression and myelosuppression, which may have confounded the observed treatment response. Second, TSAT and serum ferritin were not measured in all patients, which may have introduced selection bias. Third, the absence of a control group prevents definitive exclusion of spontaneous Hb recovery or the influence of chemotherapy scheduling; however, patients with treatment interruption were censored to minimize this potential impact. Fourth, the short observation period limited the long-term assessment of efficacy and safety. Finally, although no IV iron-related adverse events were observed, the sample size was insufficient to adequately evaluate rare complications.

## Conclusions

In this single-institution retrospective study, IV iron monotherapy showed sufficient efficacy even in patients with CIA and Hb levels below 10 g/dL. No significant differences in treatment response were observed according to TSAT or other iron-related parameters. Although this exploratory analysis was not powered to definitively evaluate the predictive value of TSAT or ferritin, the present findings suggest that, in routine clinical practice, IV iron may be considered for patients with Hb <10 g/dL without necessarily awaiting TSAT measurement, particularly in clinical settings where ESA therapy is not available. Larger prospective studies are required to validate these findings.
